# High rates of blood transfusion associated with Parkinson’s disease

**DOI:** 10.1007/s10072-022-06097-6

**Published:** 2022-05-02

**Authors:** Shane Shahrestani, Julian Gendreau, Ali R. Tafreshi, Nolan J. Brown, Khashayar Dashtipour

**Affiliations:** 1 Keck School of Medicine, University of Southern California, Los Angeles, CA USA; 2 Department of Medical Engineering, California Institute of Technology, Pasadena, CA USA; 3 Department of Biomedical Engineering, Johns Hopkins Whiting School of Engineering, Baltimore, MD USA; 4 Department of Neurological Surgery, Geisinger Health System, Danville, PA USA; 5 School of Medicine, University of California, Irvine, Irvine, CA USA; 6 Department of Neurology, Loma Linda University, Loma Linda, CA USA

**Keywords:** Parkinson’s disease, Hematology, Blood Transfusion, Gastrointestinal bleeding

## Abstract

**Background:**

As evidence continues to accumulate regarding the multi-organ dysfunction associated with Parkinson’s disease (PD), it is still unclear as to whether PD increases the risk of hematological pathology. In this study, the authors investigate the association between PD and hematological pathology risk factors.

**Methods:**

This retrospective cohort analysis was conducted using 8 years of the National Readmission Database. All individuals diagnosed with PD were queried at the time of primary admission. Readmissions, complications, and risk factors were analyzed at 30-, 90-, 180-, and 300-day intervals. Statistical analysis included multivariate Gaussian-fitted modeling using age, sex, comorbidities, and discharge weights as covariates. Coefficients of model variables were exponentiated and interpreted as odds ratios.

**Results:**

The database query yielded 1,765,800 PD patients (mean age: 76.3 ± 10.4; 44.1% female). Rates of percutaneous blood transfusion in readmitted patients at 30, 90, 180, and 300 days were found to be 8.7%, 8.6%, 8.3%, and 8.3% respectively. Those with anti-parkinsonism medication side effects at the primary admission had increased rates of gastrointestinal (GI) hemorrhage (OR: 1.02; 95%CI: 1.01–1.03, *p* < 0.0001) and blood transfusion (OR: 1.06; 95%CI: 1.05–1.08, *p* < 0.0001) at all timepoints after readmission. PD patients who experienced GI hemorrhage of any etiology, including as a side effect of anti-parkinsonism medication, were found to have significantly higher rates of blood transfusion at all timepoints (OR: 1.14; 95%CI: 1.13–1.16, *p* < 0.0001).

**Conclusions:**

Blood transfusions were found to be significantly associated with anti-parkinsonism drug side effects and GI hemorrhage of any etiology.

**Supplementary Information:**

The online version contains supplementary material available at 10.1007/s10072-022-06097-6.

## Introduction

Parkinson’s disease (PD) is the second most common neurodegenerative disease and affects almost one million people in the USA; approximately 315 out of every 100,000 people are diagnosed with PD [1–4]. PD is a recognizable syndrome diagnosed clinically with a variety of different symptoms and ancillary testing available for patients with atypical presentation [5]. Motor symptoms include akinesia, bradykinesia, tremor, rigidity, deficits of gait, deficits of speech, and deficits of handwriting [6]. Variables that are found to protect against this disease have been shown to include coffee consumption, smoking, and physical activity [7]. Risk factors for the syndrome include family history, dyspepsia, pesticide exposure, oils, and general anesthesia [7]. Non-motor symptoms include hyposmia, sleep disorders, depression, constipation, and olfactory hallucinations [8–10]. In addition, most patients with PD suffer from other comorbidities such as diabetes, ischemic and congestive heart diseases, COPD, and malignancies [11–13]. Eventually, many of these comorbidities can lead to hospital admission and even mortality in PD patients. In addition to the aforementioned comorbidities, hematological disorders have been documented as a reason for hospital admission among patients with PD [14, 15]. However, there is no large-scale investigation of the relationship between hemopathy and PD, and the direction of causality and predictive factors remain unknown.

Recent clinical studies have identified a range of blood disorders associated with PD as well as abnormalities induced by PD treatment [16–18]. In particular, anti-parkinsonian medications themselves have been associated with coagulation-fibrinolysis marker derangements, but their clinical importance has not yet been investigated thoroughly [16]. Cases of thrombocytopenia have been reported in PD patients undergoing treatment with levodopa, and this has been posited as a risk factor for GI bleeding [17, 18] The mechanism of the platelet dysfunction is thought to be autoimmune in nature, but has not been tested in a controlled fashion [18].

Gut pathology has been broadly associated with PD, and an increased prevalence of constipation and gastroparesis in PD patients has been described in prior studies [19–21]. Others have demonstrated the expression and pathology of α-synuclein in the gut, presenting a possible mechanistic model for the development of GI disturbance in PD [20, 22, 23]. Studies of PD models have demonstrated the connection of synucleinopathy in the gut to that in the brain including migration of pathology to the brain and protein misfolding in the gut that mirrors that in the brain, including a connection between PD and bowel inflammation [24–26]. The clinical manifestations and relevance to management of these significant findings are not yet clear in humans but are the topic of active investigation.

The exact causative mechanisms of this hemopathy and increased bleeding risk are not understood and are challenging to investigate. As evidence grows for the role of the multi-organ involvement associated with the development of neurodegenerative diseases, an understanding of the relationship between PD and hemopathy is of particular contemporary relevance for patient management [27, 28]. In this study, we use a nationally representative database to describe the high prevalence of blood transfusion among PD patients. Using this data, we develop predictive models and survival curves for the hematological disturbances seen in PD and present a framework for understanding its causes.

## Methods

### Data source

In this study, we use the Healthcare Cost and Utilization Project (HCUP) National Readmission Database (NRD) from the years 2010 to 2017. The NRD is a large yearly database that publishes national information regarding inpatient demographics, diagnoses, procedures, and readmissions. Patient hospital admissions are de-identified and are each represented as unique patient linkages to allow for accurate patient tracking throughout the calendar year. The NRD is publicly available for purchase and has been designed to allow for nationally representative readmission analysis when used in conjunction with the provided NRD discharge weights. Between all years of NRD included in this study, we identified more than 100 million patient discharges, and all data regarding patient diagnoses and procedures were queried using *International Classification of Diseases, Ninth Revision* (ICD-9) and *International Classification of Diseases, Tenth Revision* (ICD-10) codes in all patient admissions and readmissions. Using relevant cost-to-charge ratios (CCR) provided with the NRD, which are imputed from national hospital-specific or hospital group averaged all-payer inpatient cost data, we converted hospital charges to inpatient costs. Institutional Review Board (IRB) approval and informed consent were not required as we used a de-identified publicly available database.

#### Patient selection and analysis

All patients with a diagnosis of PD on primary admission from 2010 to 2017 were queried using relevant ICD-9 and ICD-10 coding (Supplementary Information 1) and appropriate NRD weighting, from which we identified 1,765,800 unique PD patients. These patients were pooled and the most common procedure for all patients at primary and readmission was found to be percutaneous transfusion of nonautologous packed red blood cells (RBCs) into a peripheral vein. Multi-organ conditions were queried, including Crohn’s disease, ulcerative colitis, constipation, irritable bowel syndrome (IBS), gastrointestinal (GI) bleeding, urinary tract infection (UTI), and urinary retention. Side effects of anti-parkinsonism medications (including carbidopa/levodopa and dopamine agonists) were also queried using appropriate ICD-9 and ICD-10 coding outlined in Supplementary Information 1. Charlson Comorbidity Index (CCI) scores were collected for each patient and used to develop 10-year survival estimates for all patients [29–31]. Both CCI and 10-year survival were developed by Charlson et al. in his 1987 publication and have since been validated through several studies that followed [29–33]. Readmissions and relevant complications at readmission were queried at 30-, 90-, 180-, and 300-day intervals. Patients without full follow-up were excluded at all timepoints (i.e., patients admitted in December were excluded for 30-day follow-up calculations). Patients were stratified by income quartiles based on estimated median household income by ZIP code, insurance type, hospital type, and discharge status. The Kaplan–Meier survival curves were developed to visualize the effects of patient factors on relevant complications in those readmitted within one calendar year.

#### Statistical analysis

All statistics included in this analysis were conducted in RStudio (Version 1.2.5042). All statistical tests were two-sided and used an *α* = 0.05 level of significance. The Kaplan–Meier estimation with log-rank test was used to assess for blood transfusion at readmission in patients with gastrointestinal hemorrhage and drug complications.

### Multivariate analysis

Gaussian-fitted generalized regression modeling was used to determine the effect of patient characteristics at primary admission on blood transfusion requiring or at the time of readmission. The Wald testing was performed to evaluate the effect of the weighted distance between the estimated value and the hypothesized true value under the null hypothesis on statistical parameters within each model. Coefficients of continuous variables analyzed within the multivariate models were exponentiated and interpreted as odds ratios. Independent covariates for all analyses were age, sex, CCI, and discharge weights.

### Predictive models

Univariate predictive models were developed for risk factors that were found to be significant on multivariate analysis. All predictive algorithms were developed using generalized Gaussian linear regression models and plotted to visualize the dose–response relationship between the independent variable (blood transfusion) and dependent variables of interest.

## Results

### Demographics

A total of 1,765,800 PD patients were identified at primary admission. The average age of PD patients included in this study was 76.3 ± 10.4 years, 44.1% were female, and the average CCI was 5.9 ± 2.3. Predicted average 10-year survival was found to be 19.6% in our PD cohort. The mean hospital length of stay (LOS) during primary admission was 6.3 ± 9.1 days, and the average all-payer hospital cost associated with admission in PD patients was found to be $13,256.66 ± $17,282.44. With regard to insurance type, 1,531,990 (86.8%) patients had Medicare, 54,322 (3.1%) patients had Medicaid, 139,254 (7.9%) had private insurance, and 8,318 (0.5%) were self-payers. Patients were also stratified into quartiles based on median household income by ZIP code, with 462,416 (26.2%) patients in the first (highest) quartile, 450,083 (25.5%) patients in the second quartile, 427,258 (24.2%) patients in the third quartile, and 402,208 (22.8%) patients in the fourth (lowest) quartile. Most patients were admitted to metropolitan hospitals, with 906,308 (51.3%) patients being admitted to a metropolitan teaching hospital, 628,000 (35.6%) being admitted to a metropolitan non-teaching hospital, and 231,493 (13.1%) being admitted to a non-metropolitan hospital. Within all discharges, 577,040 (32.7%) were considered routine with the remaining 1,188,760 (67.3%) being non-routine discharges including transfers to short-term hospitals, skilled nursing facilities, home healthcare facilities, discharges against medical advice, and patient death.

### Complications

Within 30, 90, 180, and 300 days of discharge, the readmission rate of PD patients was found to be 12.3%, 25.5%, 36.3%, and 45.8% respectively. The rate of percutaneous transfusion of nonautologous packed RBCs in readmitted patients at 30, 90, 180, and 300 days was found to be 8.7%, 8.6%, 8.3%, and 8.3% respectively (Table 1). At all timepoints, multivariate analysis controlling for age, sex, and comorbidities revealed that PD patients experiencing side effects from anti-parkinsonism medications had increased rates of GI hemorrhage (average OR: 1.02; 95%CI: 1.01–1.03, *p* < 0.0001) and blood transfusion (average OR: 1.06; 95%CI: 1.05–1.08, *p* < 0.0001) at readmission. PD patients who experienced GI hemorrhage of any etiology, including as a side effect of anti-parkinsonism medication, were found to have significantly higher rates of blood transfusion at all timepoints (average OR: 1.14; 95%CI: 1.13–1.16, *p* < 0.0001) (Table 2). Furthermore, PD patients who received blood transfusion procedures during readmission were found to have significantly higher readmission rates at all timepoints compared to patients readmitted for any other indication (average *p* = 0.0014).

**Table 1:** Complication rates for PD readmission cohorts

	30-day readmission (*n* = 1,630,578)	90-day readmission (*n* = 1,367,850)	180-day readmission (*n* = 979,040)	300-day readmission (*n* = 371,804)
Primary				
Constipation	157,852 (9.7%)	131,477 (9.6%)	92,266 (9.4%)	34,555 (9.3%)
IBS	16,655 (1.0%)	13,973 (1.0%)	9,736 (1.0%)	3,741 (1.0%)
Crohn’s disease	4,860 (0.3%)	4,153 (0.3%)	2,934 (0.3%)	1,141 (0.3%)
Ulcerative colitis	5,121 (0.3%)	4,262 (0.3%)	2,995 (0.3%)	1,179 (0.3%)
Gastrointestinal hemorrhage	20,931 (1.3%)	17,593 (1.3%)	13,065 (1.3%)	5,084 (1.4%)
UTI	335,252 (20.6%)	280,983 (20.5%)	201,352 (20.6%)	78,874 (21.2%)
Urinary retention	83,905 (5.1%)	69,216 (5.1%)	48,509 (5.0%)	18,248 (5.0%)
Anti-parkinsonism drug effect	20,534 (1.3%)	17,800 (1.3%)	12,945 (1.3%)	5,194 (1.4%)
Readmission				
Readmission rate	200,078 (12.3%)	349,007 (25.5%)	355,591 (36.3%)	170,424 (45.8%)
Blood transfusion	17,315 (8.7%)	30,110 (8.6%)	29,571 (8.3%)	14,146 (8.3%)

**Table 2:** Odds ratios for blood transfusion at readmission obtained from multivariate models

	Odds ratio	95% confidence interval	*p*-value
30-day readmission			
Constipation	0.99	0.99–1.00	0.09
IBS	0.99	0.98–1.01	0.55
Crohn’s disease	1.03	0.99–1.06	0.10
Ulcerative colitis	1.02	0.99–1.05	0.21
Gastrointestinal hemorrhage	1.14	1.13–1.16	< 0.0001*
UTI	1.00	0.99–1.00	0.74
Urinary retention	1.00	1.00–1.01	0.25
Anti-parkinsonism drug effect	1.06	1.04–1.08	< 0.0001*
90-day readmission			
Constipation	0.99	0.989–0.999	0.0045*
IBS	0.99	0.97–1.00	0.07
rohn’s disease	1.04	1.01–1.06	0.008*
Ulcerative colitis	1.02	1.01–1.05	0.046*
Gastrointestinal hemorrhage	1.16	1.14–1.17	< 0.0001*
UTI	1.00	1.00–1.01	0.14
Urinary retention	1.00	0.99–1.00	0.57
Anti-parkinsonism drug effect	1.07	1.06–1.09	< 0.0001*
180-day readmission			
Constipation	1.00	0.99–1.00	0.08
IBS	0.98	0.970–0.997	0.02*
Crohn’s disease	1.03	1.01–1.06	0.009*
Ulcerative colitis	1.02	0.99–1.04	0.20
Gastrointestinal hemorrhage	1.14	1.13–1.16	< 0.0001*
UTI	1.00	1.00–1.01	0.12
Urinary retention	1.00	0.99–1.00	0.27
Anti-parkinsonism drug effect	1.05	1.04–1.07	< 0.0001*
300-day readmission			
Constipation	0.99	0.985–0.998	0.008*
IBS	0.99	0.97–1.01	0.18
Crohn’s disease	1.04	1.01–1.08	0.04*
Ulcerative colitis	1.02	0.99–1.06	0.21
Gastrointestinal hemorrhage	1.12	1.11–1.14	< 0.0001*
UTI	1.00	1.00–1.01	0.66
Urinary retention	1.00	0.99–1.01	0.54
Anti-parkinsonism drug effect	1.06	1.04–1.08	< 0.0001*

* denotes statistically significant value

Several individual GI disorders were found to be significantly related with blood transfusion rates at readmission, but none of them had consistent trends at all readmission time intervals. PD patients with ulcerative colitis were found to have a higher odds of blood transfusion at only 90-day readmission (OR: 1.02; 95%CI: 1.01–1.05, *p* = 0.046) and those with Crohn’s disease were found to have a higher odds of blood transfusion at 90, 180, and 300 days (average OR: 1.04; 95%CI: 1.01–1.07, *p* = 0.02). Conversely, PD patients with constipation were found to have a lower odds of blood transfusion at 90 days (OR: 0.993; 95%CI: 0.989–0.998, *p* = 0.005) and 300 days (OR: 0.991; 95%CI: 0.985–0.998, *p* = 0.008), and those with IBS were found to have a lower odds of blood transfusion at only 180 days (OR: 0.983; 95%CI: 0.970–0.997, *p* = 0.02). Multivariate analysis revealed no significant relationship between genitourinary (GU) complications, including urinary retention and UTI, and blood transfusion at all timepoints.

### Predictive models

Predictive regression models were developed for complications found to be significantly associated with higher rates of blood transfusion at all readmission timepoints on multivariate analysis. PD patients with ICD coding for anti-parkinsonism drug side effects on primary admission were found to have an attributable 7.7%, 8.9%, 7.0%, and 7.4% increase in blood transfusion rates at 30-, 90-, 180-, and 300-day readmission respectively compared to patients without drug effects (*p* < 0.0001 for all) (Supplementary Information 2). Furthermore, patients with GI hemorrhage on primary admission were found to have an attributable 14.2%, 15.1%, 14.1%, and 12.4% increase in blood transfusion rates at 30-, 90-, 180-, and 300-day readmission respectively compared to patients without GI hemorrhage on primary admission (*p* < 0.0001 for all) (Supplementary Information 2).

### Survival analysis

**Fig. 1:**
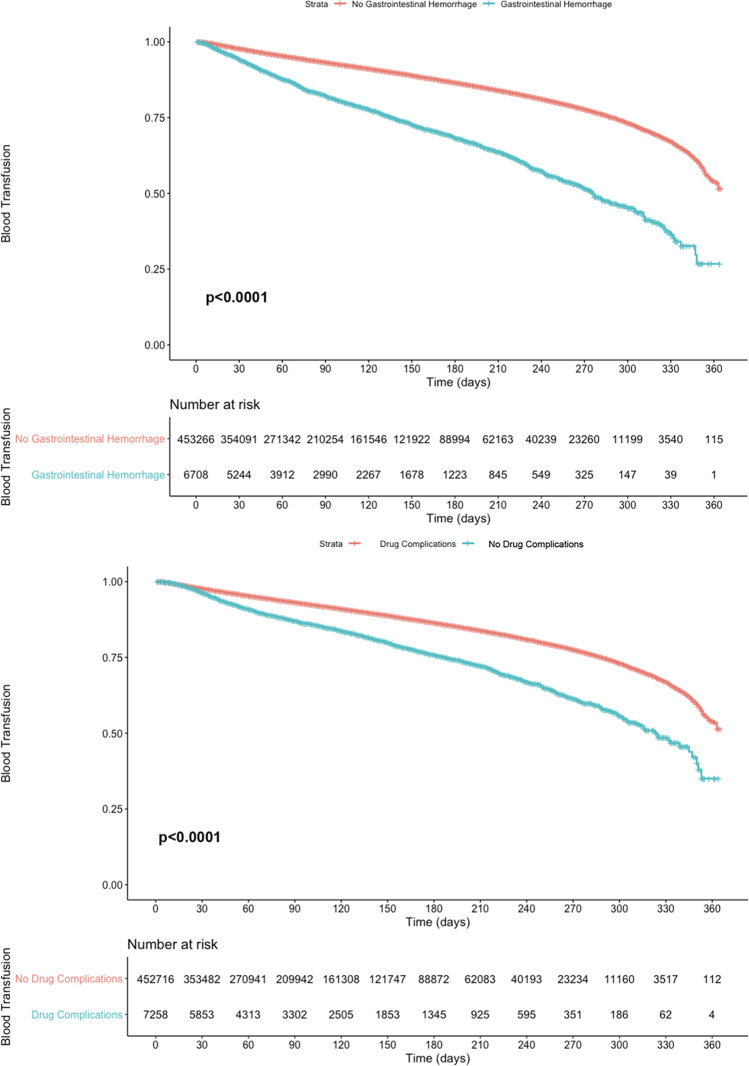
Kaplan–Meier estimation with log-rank test for blood transfusion at readmission. Increased blood transfusion was associated with gastrointestinal hemorrhage and increased drug complications (*p* < 0.0001)

The Kaplan–Meier curves were developed to visualize the trends in blood transfusion at readmission in patients with GI hemorrhage and anti-parkinsonism drug complications at primary admission. Risk tables are included for each respective condition to demonstrate the number of PD patients at risk. Overall, patients with both GI hemorrhage and anti-parkinsonism drug side effects at primary admission had significantly higher rates of blood transfusion within one calendar year compared to patients without each respective risk factor (for both) (Fig. 1).

## Discussion

In this 8-year retrospective cohort study of patients diagnosed with PD, we describe high rates of percutaneous transfusion of nonautologous packed RBCs at readmission. Because of the multi-system effects of PD, we investigated several GI and GU patient conditions that may influence the high rates of blood transfusion seen in our PD cohort. GI hemorrhage was found to be the best predictor of blood transfusion at all readmission timepoints, and inflammatory bowel diseases (IBDs) were found to increase the risk of blood transfusion at several, but not all, readmission timepoints.

The high rates of blood transfusion may be expected in PD patients with GI pathology. Albeit at varying time intervals, we describe higher rates of blood transfusion in the PD population with GI pathologies such as IBD, which themselves are associated with increased risk of bleeding [34]. The direction of causality between PD and IBD is currently unknown, but the association presents an apparent theory for the etiology of the increased need for blood transfusion in PD patients.

Literature outlining hematological complications in patients with PD is currently limited [35–37]. A recent study found that PD patients may have lower concentrations of iron in cerebrospinal fluid, suggesting that iron homeostasis may be partially disturbed in PD patients [38]. In addition, a review of neurosurgical literature revealed that PD has been associated with increased rates of acute posthemorrhagic anemia and blood loss following spine surgery [39, 40]. Although the etiology of this finding is not known, it may be explained by changes in hemostasis primarily in PD or secondary to PD medications. Reports showed that PD patients who use anti-parkinsonism medications have increased markers of fibrinolysis, including D-dimer, prothrombin time, creatine kinase, and E-selectin, compared to normal control patients [16, 39]. Other studies have described increases in inflammation and a resultant hypercoagulative state in PD [40–43]. Accordingly, we described that PD patients experiencing complications from their medications were at increased risk of needing blood transfusion within one calendar year. Although the biochemical abnormalities associated with PD medications could influence factors such as postoperative bleeding, further studies are needed to determine the cause of increased rates of transfusion or GI bleeding.

Lastly, the findings of this study may have significant implications for the management of patients diagnosed with PD. We find multiple comorbidities such as GI pathologies within PD patient cohorts that act as risk factors for readmission and blood transfusion. As a result, frequent GI and hematological screening in PD patients should be employed with hopes of reducing further complications. Further, frequent screening may allow physicians to gauge how PD patients respond to anti-parkinsonism medications, allowing for early interventions (such as lowering drug dosages) to prevent unnecessary patient readmissions. Targeted medical management and preventive counseling of PD patients can help prevent or mitigate complications from medication side effects or comorbidities which may lead to blood transfusion.

### Limitations

There are several limitations to this study. First, the retrospective nature of this database study is limited by the quality of medical coding. This is especially true as the ICD coding for anti-parkinsonism was not specific to individual medications and thus grouped all medications used for PD treatment together in one group. This limited conclusion could have been made for individual medications. Second, this study included NRD years from 2010 to 2017, during which hospital coding changed from ICD-9 to ICD-10. Although this paradigm shift in coding may introduce potential coding errors, this time span was chosen to maximize the size of our patient cohort, allowing for broad trend analysis.

## Conclusion

As more data regarding the multi-system effects of PD emerges, little is known about its clinically relevant hematological effects and GI pathologies. Further, predictive models demonstrate that PD patients with GI hemorrhage require the highest rates of blood transfusion. Additional longitudinal studies evaluating the interaction between PD, GI pathologies, and hematological pathologies are necessary to understand the association between PD and blood transfusion.

## Supplementary Information

Below is the link to the electronic supplementary material.

Supplementary file1 (DOCX 686 KB)
