# Supplementary material for: High rates of blood transfusion associated with Parkinson’s disease
Source: Neurol Sci. 2022 May 2;43(8):4761–8. doi: 10.1007/s10072-022-06097-6 (PMC9349070; doi:10.1007/s10072-022-06097-6)

**Supplementary Information 1:** ICD-9 and ICD-10 codes used in analysis.

| **Diagnosis/Procedure of Interest** | **ICD-9 \| ICD-10 Codes** |
| --- | --- |
| Parkinson’s Disease | 3320 \| G20 |
| Constipation | 56400, 56409, 56402, 56401 \| K5900, K5901, K5902, K5904, K5909 |
| Blood Transfusion | 9904 \| 30233N1 |
| Gastrointestinal Hemorrhage | 5789 \| K922 |
| Irritable Bowel Syndrome | 564, 78799, 5645 \| R194, K591, K588, K582, K580, K589 |
| Ulcerative Colitis | 5566, 5568, 5569, 5565, 5560, 5561, 5562, 5563 \| K5100, K51011, K51012, K51013, K51014, K51018, K51019, K5120, K51211, K51212, K51213, K51214, K51218, K51219, K5130, K51311, K51312, K51313, K51314, K51318, K51319, K5140, K51411, K51412, K51413, K51414, K51418, K51419, K5150, K51511, K51512, K51513, K51514, K51518, K51519, K5180, K51811, K51812, K51813, K51814, K51818, K51819, K5190, K51911, K51912, K51913, K51914, K51918, K51919 |
| Crohn’s Disease | 5550, 5551, 5552, 5559 \| K5100, K51011, K51012, K51013, K51014, K51018, K51019, K5120, K51211, K51212, K51213, K51214, K51218, K51219, K5130, K51311, K51312, K51313, K51314, K51318, K51319, K5140, K51411, K51412, K51413, K51414, K51418, K51419, K5150, K51511, K51512, K51513, K51514, K51518, K51519, K5180, K51811, K51812, K51813, K51814, K51818, K51819, K5190, K51911, K51912, K51913, K51914, K51918, K51919 |
| Anti-Parkinsonism Drug Side Effect | 966, 9664 \| T428X1A, T428X2A, T428X3A, T428X4A |

**Supplementary Information 2:** Predictive models for blood transfusion at readmission.


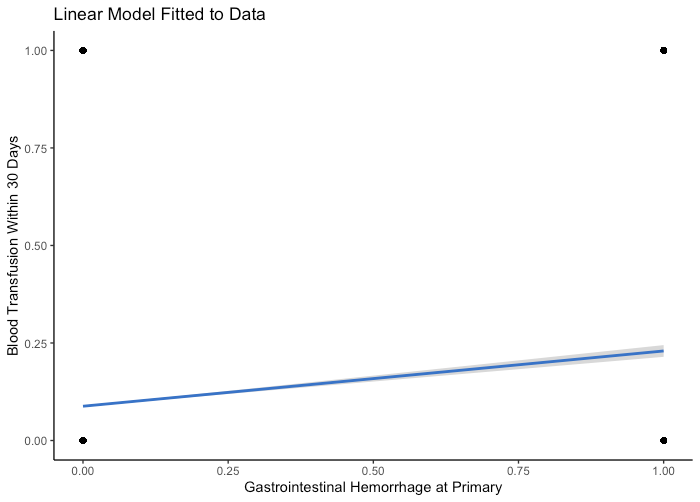

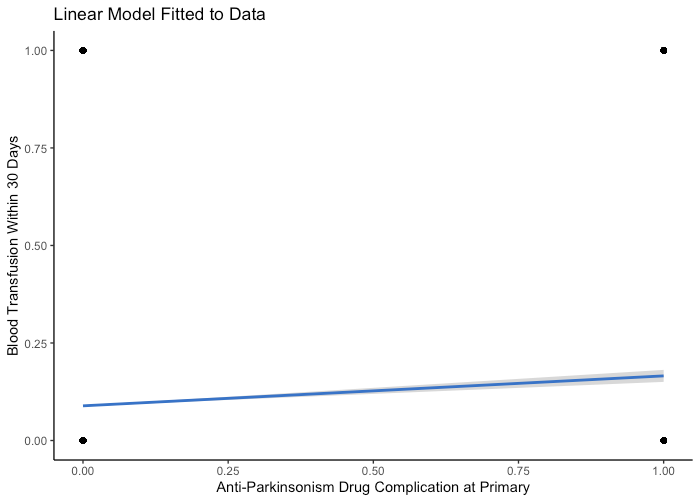

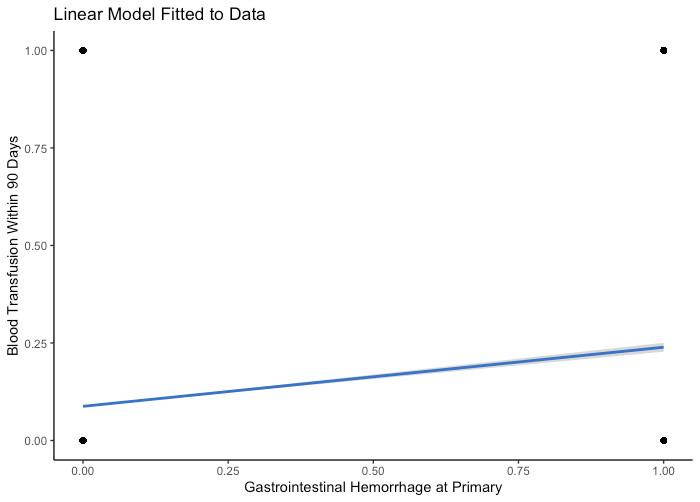

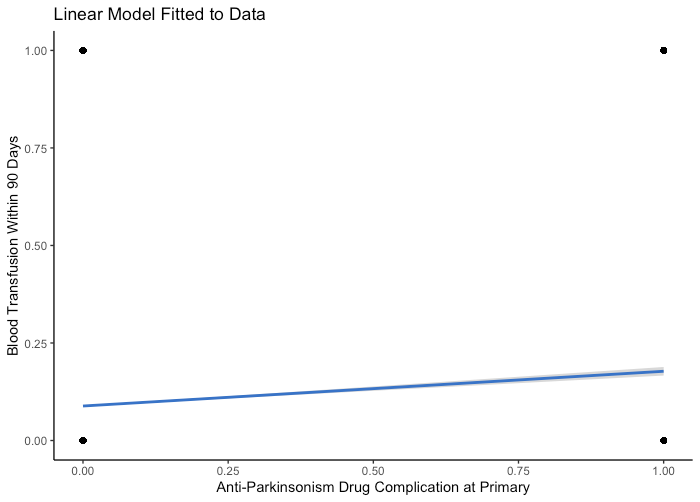

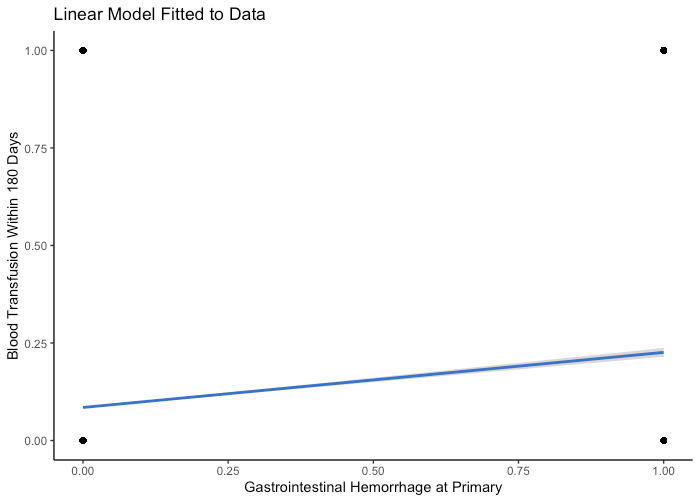

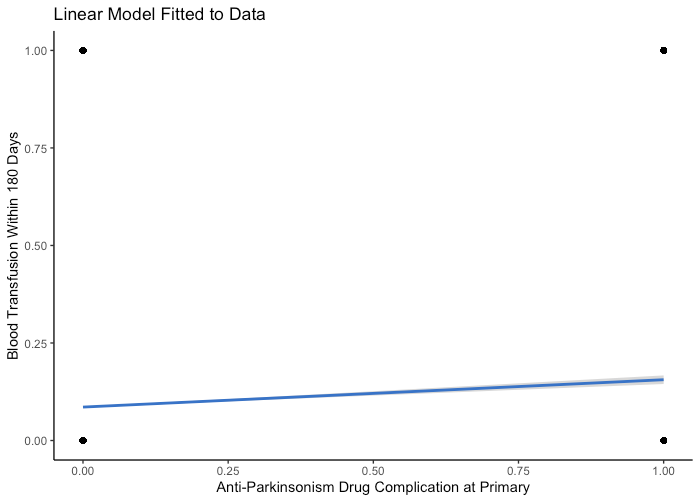

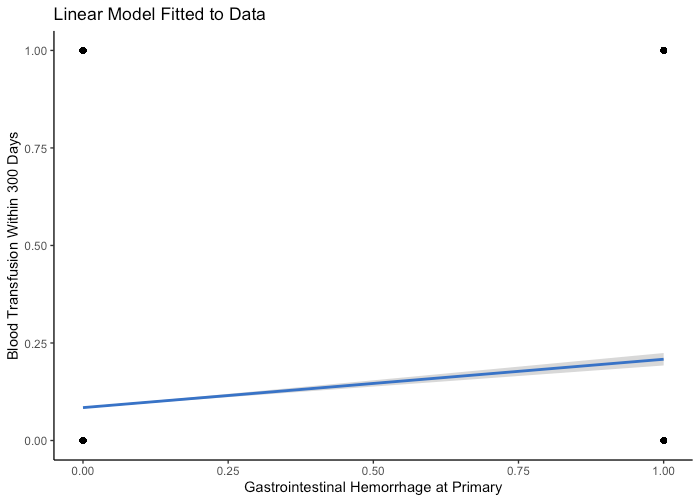

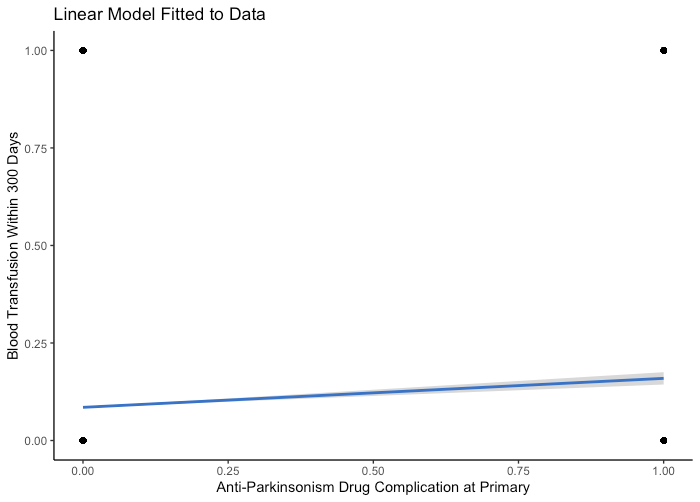

Supplement: Supplementary file 1 — Supplementary file1 (DOCX 686 KB) [file 10072_2022_6097_MOESM1_ESM.docx]
